# Reductive and Coordinative Effects of Hydrazine in Structural Transformations of Copper Hydroxide Nanoparticles

**DOI:** 10.3390/nano9101445

**Published:** 2019-10-11

**Authors:** Xenia Medvedeva, Aleksandra Vidyakina, Feng Li, Andrey Mereshchenko, Anna Klinkova

**Affiliations:** 1 Department of Chemistry and Waterloo Institute for Nanotechnology, University of Waterloo, Waterloo, ON N2L 3G1, Canada; kseniia.azarova@uwaterloo.ca (X.M.); vidyakina.aleksandra@mail.ru (A.V.); feng.li@uwaterloo.ca (F.L.); a.mereshchenko@spbu.ru (A.M.); 2 Institute of Chemistry, Saint-Petersburg State University, 7/9 Universitetskaya emb., St. Petersburg 199034, Russia

**Keywords:** copper oxide, copper hydroxide, hydrazine, structural transformation, self-assembly, crystallization by particle attachment

## Abstract

Shape-specific copper oxide nanostructures have attracted increasing attention due to their widespread applications in energy conversion, sensing, and catalysis. Advancing our understanding of structure, composition, and surface chemistry transformations in shaped copper oxide nanomaterials during changes in copper oxidation state is instrumental from both applications and preparative nanochemistry standpoints. Here, we report the study of structural and compositional evolution of amorphous copper (II) hydroxide nanoparticles under hydrazine reduction conditions that resulted in the formation of crystalline Cu_2_O and composite Cu_2_O-N_2_H_4_ branched particles. The structure of the latter was influenced by the solvent medium. We showed that hydrazine, while being a common reducing agent in nanochemistry, can not only reduce the metal ions but also coordinate to them as a bidentate ligand and thereby integrate within the lattice of a particle. In addition to shape and composition transformation of individual particles, concurrent interparticle attachment and ensemble shape evolution were induced by depleting surface stabilization of individual nanoparticles. Not only does this study provide a facile synthetic method for several copper (I) oxide structures, it also demonstrates the complex behavior of a reducing agent with multidentate coordinating ability in nanoparticle synthesis.

## 1. Introduction

Controlled fabrication of copper oxides and hydroxides (e.g., CuO, Cu_2_O, CuOH, Cu(OH)_2_) nanostructures in different morphologies has attracted increasing attention in recent years due to their potential applications in energy conversion, electrode materials, sensing, and catalysis [1,2,3]. Specifically, these materials have been used as a negative electrode material for lithium-ion batteries [4,5], a stable catalyst for photochemical water splitting [6,7], a replacement for expensive noble metal catalysts in the oxidation of carbon monoxide, nitric oxide, volatile organics [8,9,10], and in C–N and C–S cross-coupling reactions [11,12]. In addition, various copper oxide materials have been used as precursors for oxide-derived copper for CO_2_ electroreduction [13,14,15]. Maximization of their performance metrics in these processes relies on tailoring their structure, composition, and surface chemistry to optimize their physical and chemical properties that enable these applications.

To this end, variously shaped copper oxide nanostructures [16], including cubes [17], cuboctahedra [18], octahedral [19], multipods [20], nanowires [21], nanotubes [22], and hollow structures [23], have been synthesized to date. Specifically, multipod, branched, or dendric nanoparticles are of particular interest in conventional, electro-, and photocatalysis due to their (a) highly developed surface area, (b) exposed high index facets and edges with undercoordinated sides, and (c) inability to densely pack on substrates, resulting in rough and porous material deposits, with all of these factors generally leading to higher catalytic activity [24,25]. Shaped copper oxide nanostructures are typically prepared by chemical reduction, electrodeposition, and solvothermal synthesis methods. In addition to traditional solution-based methods, shape-specific copper oxide micro- and nanostructures can be obtained from shape-defined nanostructured solid precursors via chemical oxidation in the presence of shape-directing surfactants [26] as well as via an electrochemical process [27], although few reports explored this approach and associated structural changes. At the same time, only limited information is available regarding structural transformations of amorphous copper oxide nanoparticles upon inducing changes in their oxidation state [1,2,3]. Further elucidation of shape transformations of copper oxide nanoparticles upon oxidation or reduction would expand the toolbox of preparative nanochemistry and enable the development of superior functional materials for energy and catalysis. Morphological transformations of amorphous copper oxide nanomaterials are of particular interest; the absence of short-range order could allow for integration or entrapment of polydentate ligands within the material upon changes in the oxidation state [28], introducing compositional moieties similar to metal-organic coordination polymers [29,30], which represents an unexplored and promising avenue of composite materials from both synthetic and application standpoints.

Here, we report the study of structural and compositional evolution of copper (II) hydroxide nanoparticles under hydrazine reduction conditions. Nanoparticle synthesis often relies on chemical reduction, with hydrazine being one of the most common reducing reagents in nanochemistry [31]. While hydrazine is typically used solely as a reducing agent [32,33,34], this work shows that, in the excess of hydrazine, it can not only reduce the metal ions but can also integrate within the lattice of the nanoparticle, resulting in the formation of copper coordination compound–oxide composite nanoparticles. At different hydrazine–copper ratios, we observed structural evolution of amorphous Cu(OH)_2_ nanocages to crystalline Cu_2_O nanoparticles and to composite Cu_2_O-N_2_H_4_ branched nanostructures. Both single particle structural evolution and multi-particle assembly and concurrent structural evolution could be performed by controlling the amount of the stabilizing polymer engulfing the nanoparticles. Interestingly, the hydrazine-containing composite nanoparticle shape could be switched from the branched to a zigzag-like structure by simply switching the reaction solvent, which suggests that the solvent plays a shape-directing role in this process. Not only does this study provide a facile and easily scalable method for several copper (I) oxide structures, but it also demonstrates the complex behavior of a reducing agent with multidentate coordinating ability in nanoparticle synthesis.

## 2. Materials and Methods

### 2.1. Materials

Copper(II) chloride (CuCl_2_, 99.995%), polyvinylpyrrolidone (PVP, M_n_ = 55 kDa), sodium borohydride (NaBH_4_, powder, 98%), hydrazine monohydrate (N_2_H_4_ 65%, reagent grade, 98% purity), anhydrous methanol (99.5%) and ethanol (99.5%) were purchased from Sigma Aldrich (Oakville, ON, Canada).

### 2.2. Cu(OH)_2_ Precursor Synthesis

Amorphous Cu(OH)_2_ nanocages were prepared using a previously reported procedure [35]. In a typical synthesis, 40 mL of ethanol and 261 mg of PVP were placed in a 250 mL Erlenmeyer flask followed by 15 min of sonication and subsequent addition of 40 mL of 5.2 mM CuCl_2_ solution in ethanol under sonication. The resultant emerald solution was stirred for 30 min, followed by the addition of 20 mL of freshly prepared 20 mM NaBH_4_ solution in ethanol in one quick shot under vigorous stirring, resulting in the immediate color change to deep brown. The final solution was left undisturbed at room temperature for 48 h. As prepared bright-blue colloid was purified by two 15 min centrifugation cycles at 5000× *g* and subsequent redispersion of the precipitate in 20 mL of ethanol to give a final stock solution of Cu(OH)_2_ nanocages.

### 2.3. N_2_H_4_ Reduction of Cu(OH)_2_

Then, 3% solution of N_2_H_4_ in deionized water was added dropwise to 10 mL of Cu(OH)_2_ nanocages stock solution under vigorous vortexing, resulting in the solution color change to orange. Specifically, to obtain Cu_2_O and Cu_2_O-N_2_H_4_ nanoparticles, stochiometric (27 µL) and 20 times excess (530 µL) of N_2_H_4_ were used, respectively. The solution was stirred for another minute and purified by a 5 min centrifugation cycle at 5000× *g* (for electron microscopy imaging, the solution can be used without purification, giving same imaging results).

To induce simultaneous assembly and reduction of Cu(OH)_2_ nanocages in the excess of N_2_H_4_ and to subsequently form larger branched Cu_2_O-N_2_H_4_ particles, initial Cu(OH)_2_ nanocages stock solution was subjected to three additional washing cycles by centrifugation at 5000× *g* and redispersion in the same amount of ethanol, otherwise the N_2_H_4_ reduction procedure remained the same.

To obtain zigzag-like structures instead of branched structures upon five times washed Cu(OH)_2_ nanocage reduction with excess hydrazine, the reaction solvent was switched from ethanol to methanol by using it for redispersion of the particles during their washing.

### 2.4. Electrochemical Reduction of Cu_2_O and Cu_2_O-N_2_H_4_

The electroreduction experiments were performed in a one-compartment cell using a three-electrode system connected to an electrochemical workstation (Biologic SP-300, purchased from SnowHouse Solutions, Lac-Beauport, QC, Canada). As prepared colloids were diluted 10 times with ethanol and dropcasted onto carbon paper strips (Toray TGP-H-060, Fuel Cell Store, College Station, TX, USA) that were used as working electrodes. Ag/AgCl (with saturated KCl as a filling solution) and platinum gauze were used as a reference and counter electrodes, respectively. The electrolyte was 0.5M KHCO_3_ buffer (pH = 7.2) saturated with argon. Four cyclic voltammetry (CV) cycles from 0 to −1.2 V vs. reversible hydrogen electr0ode (RHE) at 20 mV/s scan rate were performed to yield the electroreduced structures.

### 2.5. Nanoparticle Characterization

For the structural analysis, scanning and transmission electron microscopy (SEM and TEM, respectively) imaging was performed using a scanning transmission electron microscope Hitachi S-5200 microscope (purchased from Scanservice Corp., Tustin, CA, USA) equipped with SE and STEM detectors operating at 25 kV. Samples for imaging were prepared by depositing a droplet of the colloid on a 400 mesh carbon-coated copper grid and allowing the solvent to evaporate.

For the composition and surface analysis, concentrated, dried, and powdered samples were analyzed by X-ray photoelectron spectrometry (XPS), X-ray diffraction (XRD), elemental analysis, and infrared spectrometry using ThermoFisher Scientific K-Alpha X-ray photoelectron spectrometer (Ontario Centre for the Characterisation of Advanced Materials, Toronto, ON, Canada), Bruker D8 Advance powder X-ray diffractometer (Watlab, Waterloo, ON, Canada), Flash 2000 CHNS Analyzer (Analest, Toronto, ON, Canada), and ThermoScientific Nicolet 6700 FT-IR spectrometer (Smith Lab, Waterloo, ON, Canada) operating in transmission mode, respectively.

## 3. Results and Discussion

### 3.1. Synthesis of the Starting Material

**Figure 1:**
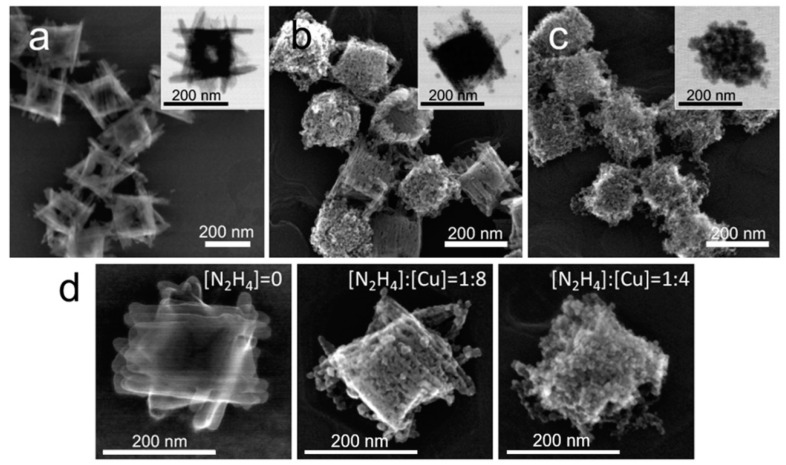
Structural evolution of Cu(OH)_2_ particles during the reduction with a stoichiometric hydrazine–copper ratio. SEM and TEM images of Cu(OH)_2_ nanocages (**a**) and reduction products obtained at [N_2_H_4_]:[Cu] = 1:8 (**b**) and at [N_2_H_4_]:[Cu] = 1:4, which corresponded to the stoichiometric hydrazine–copper ratio (**c**). TEM and high magnification SEM images of the corresponding structures are shown in the insets in (**a**–**c**) and in (**d**), respectively.

The starting material, amorphous Cu(OH)_2_ nanocages with a narrow size distribution and a regular shape, were synthesized according to a previously reported method based on the reduction of CuCl_2_ with NaBH_4_ in ethanol in the presence of PVP as a stabilizing polydentate ligand [35]. In this method, the formation of Cu(OH)_2_ nanocages proceeded through the oxidation of initially formed copper clusters. SEM and TEM images of the resultant Cu(OH)_2_ nanocages with average core size 165 ± 10 nm and extending 51 ± 17 nm arms are shown in Figure 1a and Figure S1. Elongated 17 ± 4 nm-wide bundles comprising the structure observed in the SEM and the TEM images were formed due to the anisotropic nature of Cu(OH)_2_, as it consists of complex chains formed through coordination of OH^−^ and Cu^2+^, while the bundle organization and the overall size of the particles were dictated by the stabilizing surfactant [36].

### 3.2. Structural Evolution at a Stochiometric N_2_H_4_ to Cu Ratio

During typical reduction with hydrazine, each molecule of N_2_H_4_ provides four electrons, leaving nitrogen gas and protons as byproducts. Thus, to reduce all Cu(II) to Cu(I) in Cu(OH)_2_ nanocages solution, ¼ equivalent of N_2_H_4_ was required stoichiometrically. Upon introducing this amount of the reductant to the Cu(OH)_2_ nanocage solution, an instantaneous change in color from bright blue to orange was observed (Figure S2), and the resultant particles were imaged within 5 min from the reaction using high resolution scanning transmission electron microscope. As evident from SEM and TEM images (Figure 1c), the final particle shape was reminiscent of the original nanocage shape but appeared granular, perforated, and without arm definition. When only half the stochiometric amount of N_2_H_4_ was introduced to the original solution of Cu(OH)_2_ nanocages, the SEM and the TEM image analysis clearly showed that the resultant particles were not fully reduced, with some of them being more reduced than others (Figure 1b). This uneven reduction of the particle population was likely due to mass transfer limitations, as the reduction process was extremely fast. Figure 1d shows a sequence of representative high magnification SEM images of original and reduced nanostructures at different N_2_H_4_ to copper ratios. These images show that, upon hydrazine reduction, the nanostructure did not significantly reconstruct but instead preserved the three-dimensional footprint of the original particle while losing a fraction of volume, which resulted in perforations. Interestingly, this situation only applies unless an excess of hydrazine is introduced to the particles.

### 3.3. Structural Evolution in Excess N_2_H_4_

**Figure 2:**
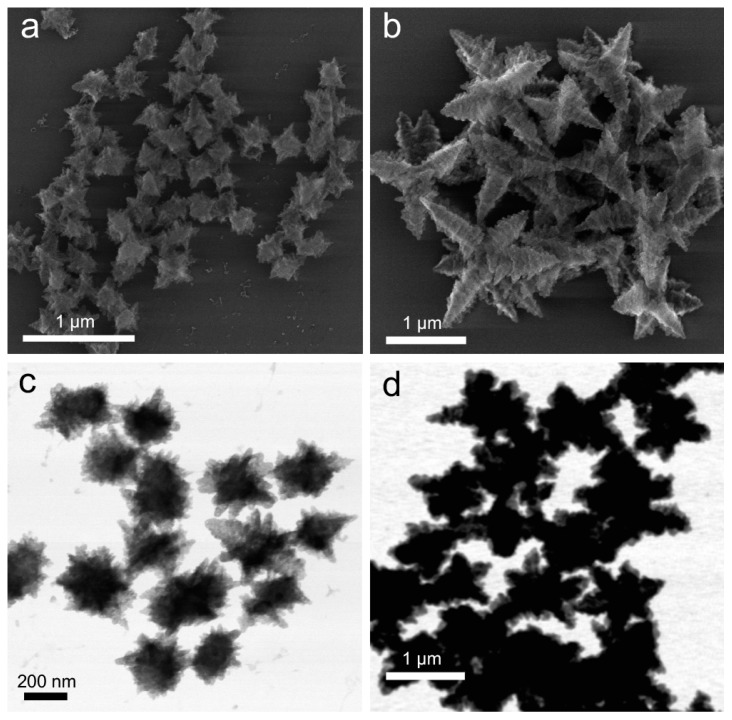
Structures obtained from Cu(OH)_2_ particles upon their reduction with excess hydrazine (20-fold compared to the stoichiometric ratio). SEM (**a**,**b**) and TEM (**c**,**d**) images of the particles obtained in the single-particle transformation regime (**a**,**c**) and in the particle attachment regime (**b**,**d**).

A 20-fold excess of hydrazine introduced to Cu(OH)_2_ nanocages purified according to the standard protocol resulted in a drastic change of the particle shape. Figure 2a,c shows SEM and TEM images of the resultant branched particles with an average size of 256 ± 35 nm, which was comparable to the size of the original nanocages, indicating that each of these particles originated from individual Cu(OH)_2_ nanocages or single-particle transformation regime.

However, when a 20-fold excess of hydrazine was introduced to extensively washed Cu(OH)_2_ nanocages, the resultant branched particles were substantially larger in size (ranging from 250 nm up to 2 µm) and had a large size variation (Figure 2b,d). This increase in size suggests that extensive removal of PVP ligands enabled interparticle surface interaction and their subsequent association coinciding with the structural transition. Considering the presence of the excess hydrazine during the reaction and the accessibility of the particle surface due to the lack of PVP, interparticle attachment could be induced by hydrazine acting as a linker between particles. Indeed, due to the presence of lone pairs on both nitrogen atoms in N_2_H_4_, hydrazine could behave as a bidentate ligand, and a number of coordination compounds of Cu(II) and Cu(I) containing hydrazine were reported in earlier literature [37,38,39]. Thus, particle attachment regime was the first indication of excess hydrazine integrating into the final structure. A further compositional analysis is provided in the following section.

The structures obtained in this particle attachment regime exhibited predominantly octahedron-derived hexapod shape, which has been observed for Cu_2_O nanoparticles [40]. However, in our case, the pods of some hexapods were not symmetrically distributed around the particle center, and their surface was very rough—a morphology that has not been previously reported for Cu_2_O. These branched particles degraded during imaging under continuous electron beam exposure (Figure S3). In addition, while being structurally stable in air for many hours, after two days, the solution darkened (Figure S4), and dense particles mixed with the original branched structures were observed in SEM and TEM (Figures S5 and S6). These denser particles were metal copper (confirmed by XRD, Figure S7), indicating that the remaining hydrazine was capable of slowly reducing Cu(I) further to Cu(0) over time.

**Figure 3:**
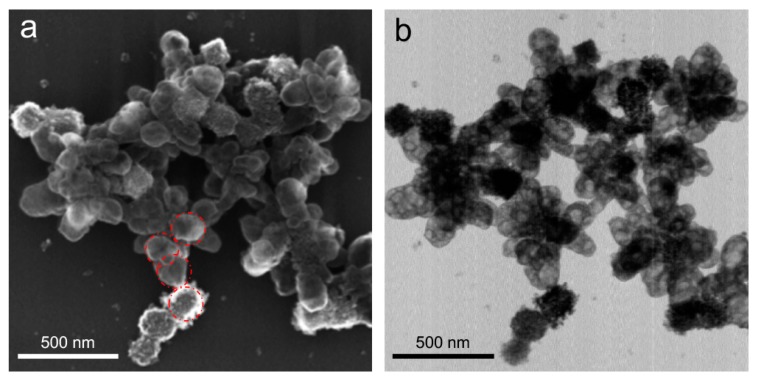
Structures obtained from Cu(OH)_2_ nanocages upon their reduction with 4-fold excess hydrazine compared to the stoichiometric ratio. SEM (**a**) and TEM (**b**) images correspond to the same sample area. Red circles in (**a**) correspond to the average size of Cu(OH)_2_ cores and are shown for eye guidance and comparison.

To gain further insight into the structural evolution in the particle attachment regime [28] in the excess hydrazine, we analyzed the structures obtained at its 4-, 5-, 10-, 15-, 20-, 50-, and 100-fold excess compared to the stochiometric ratio. At 4-fold excess hydrazine, we were able to capture intermediate structures consisting of granular and perforated Cu_2_O mixed with less electron-dense material and composed of smaller particles with sizes comparable to those of the precursor Cu(OH)_2_ nanocages (Figure 3). The lower electron density of the material observed in TEM (Figure 3b) indicated [41] that the spacing between copper atoms was increased compared to Cu_2_O, which could be explained by coordinative integration of hydrazine within the material. A patchy distribution of electron density suggested that such integration could be uneven throughout the particles (including partial transformation to metal copper, see Section 3.4 for further discussion on the composition evolution), which matches the void-containing nature of the precursor Cu(OH)_2_ nanocages and stoichiometrically reduced Cu_2_O.

**Figure 4:**
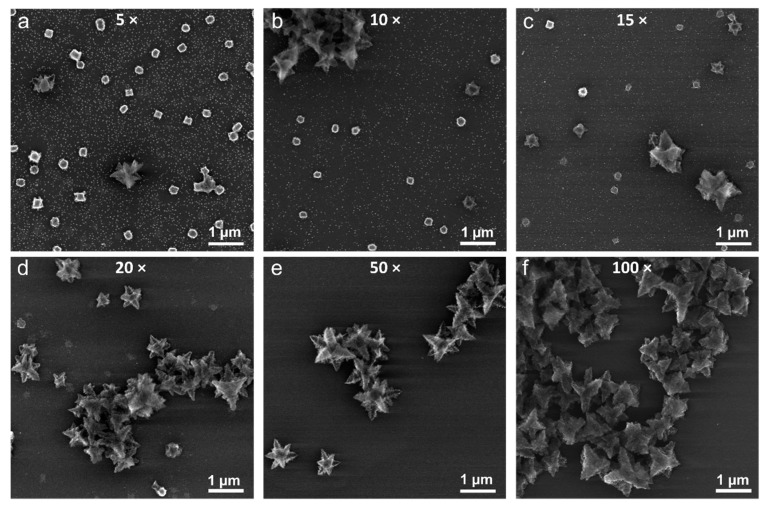
SEM images of the structures obtained from Cu(OH)_2_ particles upon their reduction with 5-fold (**a**), 10-fold (**b**), 15-fold (**c**), 20-fold (**d**), 50-fold (**e**), and 100-fold (**f**) excess hydrazine compared to the stoichiometric ratio.

The structural evolution of the reaction mixture at higher excess of hydrazine is presented in Figure 4. Granular and perforated Cu_2_O particles along with the small (sub-20 nm) nanoparticles could be found in the mixture with up to 15-fold N_2_H_4_ excess (Figure 4a–c and Figure S8). The arms of the nanocages (see inset in Figure 1a) breaking off during the initial reduction of Cu(OH)_2_ bundles could explain the origin of the small nanoparticles. The presence of small and large branched particles along with the particles noted above pointed at the rate of hydrazine incorporation being comparable with that of Cu(II) reduction, which enabled a simultaneous formation of these structures. Interestingly, the structures appeared to have approached a stable shape around 20-fold excess N_2_H_4_ (Figure 4d), as no significant structural changes or particle size increases were observed at above this hydrazine–copper ratio (Figure 4e,f and Figure S9).

### 3.4. Composition Evolution

**Figure 5:**
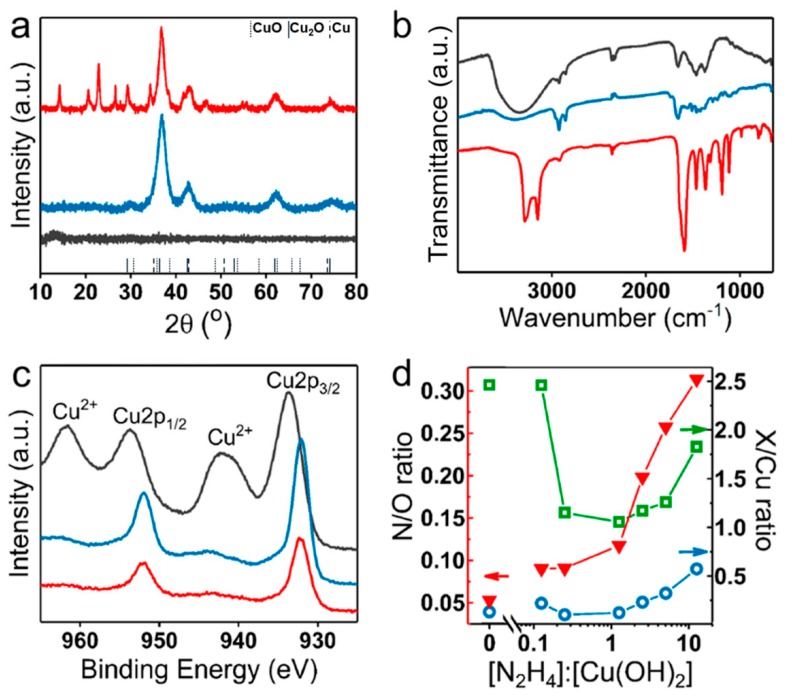
Composition characterization of Cu(OH)_2_ nanocages and the structures obtained upon their treatment with hydrazine. XRD patterns (**a**), infrared spectra (**b**), and XPS spectra of the initial Cu(OH)_2_ nanocages (black traces), reduced structures obtained at a stoichiometric hydrazine–copper ratio (blue traces) and at 20-fold excess hydrazine (red traces) (**c**). (**d**) N/O (red triangles), O/Cu (green squares), and N/Cu (blue circles) ratios obtained from quantitative X-ray photoelectron spectrometry (XPS) analysis of Cu(OH)_2_ nanocages and products obtained from their treatment with different [N_2_H_4_]:[Cu(OH)_2_] molar ratios (with [N_2_H_4_]:[Cu(OH)_2_] = 0.25 being the stoichiometric ratio).

The crystallinity of the structures was assessed using XRD (Figure 5a), and while the initial Cu(OH)_2_ nanocages were amorphous, the particles obtained at the stochiometric hydrazine–copper ratio had a typical pattern of Cu_2_O with peak broadening characteristic of nanoparticles. However, the material obtained at 20-fold excess N_2_H_4_ showed several additional peaks to those of Cu_2_O, which were indicative of a mixed phase containing Cu_2_O. Another phase was likely a coordination compound containing N_2_H_4_ as a Cu(I) ligand.

Infrared spectra of Cu(OH)_2_ nanocages and reduced particles obtained at the stoichiometric and the excess amounts of hydrazine are shown in Figure 5b. A strong broad peak at 3000–3600 cm^−1^ characteristic of O–H stretching [42] present in the initial Cu(OH)_2_ drastically decreased in the reduced structures, indicating a significant decrease in the number of OH groups. Both Cu(OH)_2_ and Cu_2_O obtained at a stochiometric hydrazine–copper ratio showed moderate C=O and C–N stretching and C–H bending peaks between 1660 and 1250 cm^−1^ as well as C–H stretching peaks between 2850 and 2950 cm^−1^ corresponding to the functional groups in PVP, present in all of these samples. Particles obtained in 20-fold excess hydrazine showed additional peaks at 3150–3350 cm^−1^ and 1600 cm^−1^, corresponding to N–H stretching and bending, respectively. This result further supported the integration of N_2_H_4_ within the final structures.

XPS analysis confirmed the reduction of Cu(II) to Cu(I) in Cu(OH)_2_ nanocages upon introduction of the stochiometric amount of hydrazine (Figure 5c). At 20-fold excess hydrazine, the XPS spectrum remained unchanged, and the satellite peak at 944 eV characteristic of Cu(I) was still present. The XPS analysis of the structures obtained at different hydrazine excess (from 0- to 50-fold) provided the information about the hydrazine uptake within the structure. Specifically, Figure 5d shows nitrogen–oxygen, nitrogen–copper, and oxygen–copper ratios as a function of the amount of introduced hydrazine obtained from the quantitative analysis of the characteristic peaks of these elements in the samples. At hydrazine–copper ratio above stoichiometric (or molar ratio above [N_2_H_4_]:[Cu(OH)_2_] = 0.25), a significant increase in N–O and N–Cu ratios was observed, in agreement with hydrazine integration within the structure. At equimolar hydrazine–copper ratio, a drastic drop in O–Cu ratio was observed, indicative of a nearly complete Cu(II) to Cu(I) reduction, while at a slight excess of hydrazine, O–Cu ratio continued to decrease. With further hydrazine–copper ratio increase, the content of oxygen in the samples began to increase, suggesting a possible solvent incorporation within the structure along with hydrazine.

According to the elemental analysis (see Supplementary Materials, Figures S12, 13, and Tables S1–S3 for details of the composition calculations), at 20-fold excess hydrazine, its NH_2_ groups were only coordinated to approximately 7–9% of Cu ions on average throughout the sample, while ethanol was coordinated to ~10% of Cu ions. This result demonstrated that the fraction of Cu_2_O phase dominated over the hydrazine-containing coordination compound, in agreement with XRD and XPS data. In addition, it pointed at the significant incorporation of the solvent within the structure under conditions favoring the hydrazine coordination.

**Figure 6:**
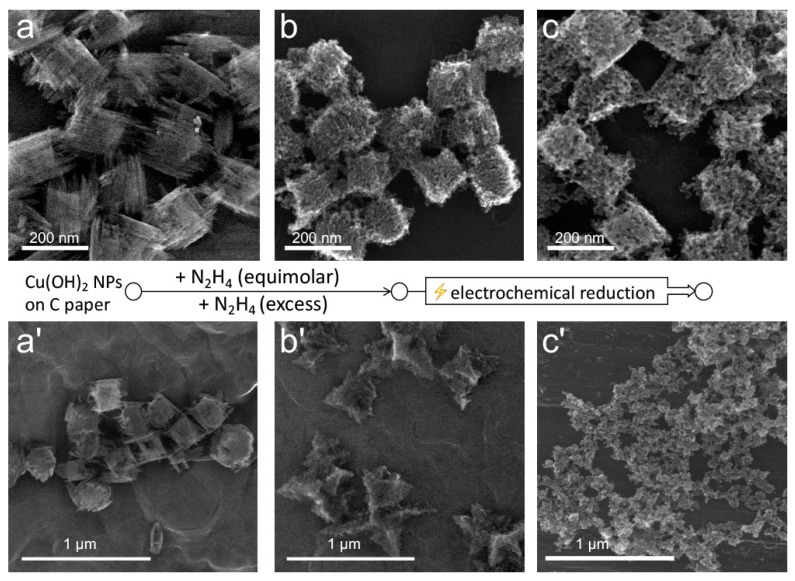
SEM images of Cu(OH)_2_ nanocages dropcasted on a carbon paper substrates (**a**,**a’**) that were treated with stoichiometric (**b**) and 20-fold excess (**b’**) amounts of hydrazine and subsequently electroreduced using four cathodic cyclic voltammetry (CV) cycles (**c**,**c’**).

To gain further insight into the structural integrity and the distribution of the two phases within the particles, we performed electrochemical reduction of the structures obtained at stoichiometric and excess amounts of hydrazine. Specifically, the hydrazine treatment was performed directly on the particles tethered to a carbon paper substrate used for the subsequent electrochemical reduction. To obtain tethered particles, Cu(OH)_2_ nanocages solution was dropcasted and dried on carbon paper strips (Figure 6a,a’), which were then dipped into the solution of hydrazine with corresponding N_2_H_4_ amounts. The obtained tethered structures are shown in Figure 6b,b’. Upon electrochemical reduction under the same cathodic conditions, the particles obtained at the stoichiometric hydrazine–copper ratio primarily preserved their overall shape while losing a fraction of their mass, which was evident from the increased size of the perforations (Figure 6c). In contrast, the particles obtained at hydrazine excess completely disintegrated, and only shape-undefined nanoporous copper deposits could be observed in their place (Figure 6c’). These deposits contained clumps of copper under 50 nm in size, with pores corresponding to those of the particles observed in Figure 6c. This result suggests that the Cu_2_O phase existed in the unreduced structures in the form of sub-50 nm granules, which were interconnected with the hydrazine-based complex of copper, assuming that the latter was easily destroyed during the electrochemical reduction.

### 3.5. Solvent Effect

**Figure 7:**
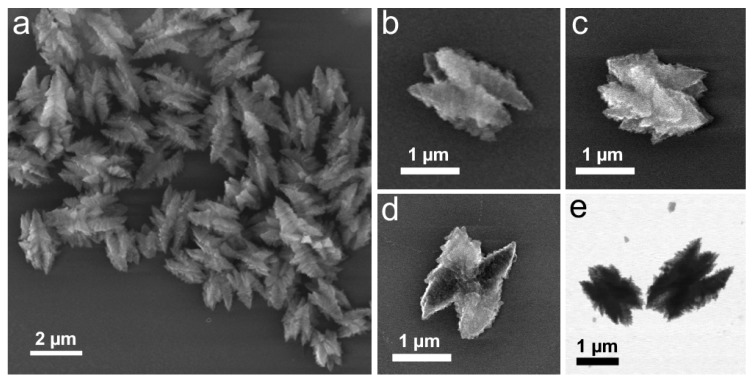
SEM (**a**–**d**) and TEM (**e**) images of the structures obtained by the reduction of Cu(OH)_2_ nanocages with excess hydrazine ([N_2_H_4_]:[Cu(OH)_2_] = 5) in methanol.

The integration of the solvent within the structure in the excess of hydrazine revealed in the compositional analysis of the materials motivated us to explore the effect of the solvent on the structural transformation of Cu(OH)_2_ nanocages. To this end, we performed their reduction with 20-fold excess hydrazine ([N_2_H_4_]:[Cu(OH)_2_] = 5) in methanol in place of ethanol as a solvent. The obtained structures were consistently of zigzag-like shape and in 1–2 µm range (Figure 7), regardless of the number of washing cycles (2–5) used to purify the initial Cu(OH)_2_ nanocages and exchange their solvent to methanol. This was likely due to higher solubility of PVP in methanol compared to ethanol [43] and subsequent surface exposure of the particles, which enabled their interparticle assembly via hydrazine linking. Indeed, when the reaction was performed in 1- or 2-propanol (slightly worth solvents for PVP compared to ethanol) [39] under the same conditions otherwise, the obtained structures matched the initial nanocages in size and showed little shape definition (Figure S10). When the same reaction was performed in water as a solvent instead of alcohols, dense Cu_2_O crystallites were observed upon imaging (Figure S11), further highlighting the shape-directing importance of the presence of alcohols in these reactions.

While the mechanism of shape selectivity in Cu(OH)_2_ reduction in excess hydrazine remains unclear and requires further investigation, our results suggest that the reaction solvent has a shape-directing effect, either via surface or bulk interaction with the transforming particle.

## 4. Conclusions

This study provides a new synthetic approach to complex copper (I) oxide particles’ branched and zigzag-like structures and describes reductive and coordinative effects of hydrazine when its excess is used for copper hydroxide reduction. More generally, we demonstrated that (1) hydrazine, while being a common reducing agent, also has coordinative ability that needs to be considered in nanochemistry reactions, especially if excess hydrazine is used; (2) when polydentate ligating species are present in the reaction medium during particle transformation, crystallization by particle attachment can take place, resulting in the addition of higher-order colloidal species (as opposed to solution-state precursors) to fully formed particles; (3) during reduction of Cu(OH)_2_ with excess hydrazine, not only hydrazine itself but also the solvent species are capable of coordinative integration within the lattice of the formed particles. In addition, these findings open the door for exploring this type of concurrent reductive/coordinative chemistry in other transition metal systems with multiple stable oxidation states.

## Supplementary Materials

The following are available online at https://www.mdpi.com/2079-4991/9/10/1445/s1, Figure S1: TEM image of as synthesized Cu(OH)_2_ nanocages, Figure S2: A photograph of Cu(OH)_2_ nanocages colloid before (left) and after (right) introducing N_2_H_4_. Note that the color of hydrazine-treated solutions was the same at a stoichiometric N_2_H_4_ amount and excess N_2_H_4_ added to Cu(OH)_2_ nanocages, Figure S3: TEM image of Cu_2_O-N_2_H_4_ obtained at [N_2_H_4_]:[Cu(OH)_2_] = 5 at 25 kV accelerating voltage at 15,000× magnification. Inset shows the effect of electron beam exposure obtained at 25 kV accelerating voltage and 70,000× magnification after 30 s exposure time (scale in the inset matches the figure), Figure S4: A photograph of Cu_2_O-N_2_H_4_ colloid (obtained by treating Cu(OH)_2_ nanocages in ethanol with hydrazine ([N_2_H_4_]:[Cu(OH)_2_] = 5) immediately after hydrazine addition (left) and after aging the mixture for 2 days (right), Figure S5: TEM (top left) and SEM (top right and bottom) images of Cu_2_O-N_2_H_4_ colloid (obtained by treating Cu(OH)_2_ nanocages in ethanol with hydrazine ([N_2_H_4_]:[Cu(OH)_2_] = 5) after its aging for 2 days, Figure S6: TEM (left) and SEM (right) images of Cu_2_O-N_2_H_4_ colloid (obtained by treating Cu(OH)_2_ nanocages in ethanol with hydrazine ([N_2_H_4_]:[Cu(OH)_2_] = 5) after its aging for 5 days, Figure S7: XRD patterns of Cu_2_O-N_2_H_4_ colloid (obtained by treating Cu(OH)_2_ nanocages in ethanol with hydrazine ([N_2_H_4_]:[Cu(OH)_2_] = 5) within a day of hydrazine addition (red) and after aging the mixture for 5 days (green), Figure S8: SEM images of the intermediate structures observed upon treatment of Cu(OH)_2_ with 5-fold excess hydrazine compared to the stoichiometric ratio ([N_2_H_4_]:[Cu(OH)_2_] = 1.25), Figure S9: TEM image of the structures obtained from Cu(OH)_2_ upon their reduction with 100-fold excess hydrazine compared to the stoichiometric ratio ([N_2_H_4_]:[Cu(OH)_2_] = 25), Figure S10: SEM images of the structures obtained by the reduction of Cu(OH)_2_ nanocages with excess hydrazine ([N_2_H_4_]:[Cu(OH)_2_] = 5) in 1-propanol (a,b) and in 2-propanol (c,d), Figure S11: SEM images of the structures obtained by the reduction of Cu(OH)_2_ nanocages with excess hydrazine ([N_2_H_4_]:[Cu(OH)_2_] = 5) in water, Figure S12: The composition of Cu(OH)_2_ nanocages and the structures obtained upon their reduction with a stoichiometric amount and 20-fold excess of hydrazine ([N_2_H_4_]:[Cu(OH)_2_] 0, 0.25, and 5, respectively). (a) Atomic fractions of H, C, and N from CHN derived from the elemental analysis. (b) Molar fractions of the molecular components calculated from the elemental analysis assuming (1) the samples contained only Cu(OH)_2_, CuO/Cu_2_O, PVP, EtOH and N_2_H_4_, and (2) all three studied samples had the same amount of PVP (i.e., no loss if PVP upon hydrazine reduction), Figure S13: Comparison of the atomic ratios (N/O, N/Cu and O/Cu), calculated from the XPS and CHN analyses’ data for the initial Cu(OH)_2_ nanocages and structures obtained by the reduction thereof with stoichiometric and excessive amounts of hydrazine ([N_2_H_4_]:[Cu(OH)_2_] 0.25 and 5, respectively), Table S1: Elemental analysis data: weight percentages (ω%) of N, C, and H in Cu(OH)_2_ nanocages and structures obtained by their reduction with a stoichiometric amount and the excess of hydrazine ([N_2_H_4_]:[Cu(OH)_2_] 0, 0.25, and 5, respectively), Table S2: Elemental analysis data converted to atomic percentages (a%) of N, C, and H, and calculated molar fractions (in %) of the components in Cu(OH)_2_ nanocages and structures obtained by their reduction with a stoichiometric amount and the excess of hydrazine ([N_2_H_4_]:[Cu(OH)_2_] 0, 0.25, and 5, respectively), Table S3: Comparison of atomic percentages (a%) of Cu, O, and N, obtained from XPS analysis and calculated from the CHN data for Cu(OH)_2_ nanocages and the structures obtained upon their reduction with a stoichiometric amount and 20-fold excess of hydrazine ([N_2_H_4_]:[Cu(OH)_2_] 0, 0.25, and 5, respectively).

## References

[1] Gawande M.B., Goswami A., Felpin F.-X., Asefa T., Huang X., Silva R., Zou X., Zboril R., Varma R.S. (2016). Cu and Cu-Based Nanoparticles: Synthesis and Applications in Catalysis. Chem. Rev..

[2] Zhang Q., Zhang K., Xu D., Yang G., Huang H., Nie F., Liu C., Yang S. (2014). CuO nanostructures: Synthesis, characterization, growth mechanisms, fundamental properties, and applications. Prog. Mater. Sci..

[3] Kuo C.-H., Huang M.H. (2010). Morphologically controlled synthesis of Cu_2_O nanocrystals and their properties. Nano Today.

[4] Park J.C., Kim J., Kwon H., Song H. (2009). Gram-Scale Synthesis of Cu_2_O Nanocubes and Subsequent Oxidation to CuO Hollow Nanostructures for Lithium-Ion Battery Anode Materials. Adv. Mater..

[5] Paolella A., Brescia R., Prato M., Povia M., Marras S., Trizio L.D., Falqui A., Manna L., George C. (2013). Colloidal Synthesis of Cuprite (Cu_2_O) Octahedral Nanocrystals and Their Electrochemical Lithiation. ACS Appl. Mater. Interfaces.

[6] Paracchino A., Laporte V., Sivula K., Gratzel M., Thimsen E. (2011). Highly active oxide photocathode for photoelectrochemical water reduction. Nat. Mater..

[7] Bornoz P., Abdi F.F., Tilley S.D., Dam B., van de Krol R., Graetzel M., Sivula K. (2014). A Bismuth Vanadate–Cuprous Oxide Tandem Cell for Overall Solar Water Splitting. J. Phys. Chem. C.

[8] Zhou K., Wang R., Xu B., Li Y. (2006). Synthesis, characterization and catalytic properties of CuO nanocrystals with various shapes. Nanotechnology.

[9] Liu Y., Fu Q., Flytzani-Stephanopoulos M. (2004). Preferential oxidation of CO in H_2_ over CuO-CeO_2_ catalysts. Catal. Today.

[10] Xu L., Sithambaram S., Zhang Y., Chen C.-H., Jin L., Joesten R., Suib S.L. (2009). Novel Urchin-like CuO Synthesized by a Facile Reflux Method with Efficient Olefin Epoxidation Catalytic Performance. Chem. Mater..

[11] Xu H.-J., Zhao X.-Y., Deng J., Fu Y., Feng Y.-S. (2009). Efficient C–S cross coupling catalyzed by Cu_2_O. Tetrahedron Lett..

[12] Rout L., Sen T.K., Punniyamurthy T. (2007). Efficient CuO-Nanoparticle-Catalyzed C–S Cross-Coupling of Thiols with Iodobenzene. Angew. Chem. Int. Ed..

[13] Li C.W., Ciston J., Kanan M.W. (2014). Electroreduction of carbon monoxide to liquid fuel on oxide-derived nanocrystalline copper. Nature.

[14] Eilert A., Cavalca F., Roberts F.S., Osterwalder J., Liu C., Favaro M., Crumlin E.J., Ogasawara H., Friebel D., Pettersson L.G.M. (2017). Subsurface Oxygen in Oxide-Derived Copper Electrocatalysts for Carbon Dioxide Reduction. J. Phys. Chem. Lett..

[15] Kas R., Kortlever R., Milbrat A., Koper M.T.M., Mul G., Baltrusaitis J. (2014). Electrochemical CO_2_ reduction on Cu_2_O-derived copper nanoparticles: Controlling the catalytic selectivity of hydrocarbons. Phys. Chem. Chem. Phys..

[16] Lignier P., Bellabarba R., Tooze R.P. (2012). Scalable strategies for the synthesis of well-defined copper metal and oxide nanocrystals. Chem. Soc. Rev..

[17] Kuo C.-H., Huang M.H. (2008). Facile Synthesis of Cu_2_O Nanocrystals with Systematic Shape Evolution from Cubic to Octahedral Structures. J. Phys. Chem. C.

[18] Ho J.-Y., Huang M.H. (2009). Synthesis of Submicrometer-Sized Cu_2_O Crystals with Morphological Evolution from Cubic to Hexapod Structures and Their Comparative Photocatalytic Activity. J. Phys. Chem. C.

[19] Xu H., Wang W., Zhu W. (2006). Shape Evolution and Size-Controllable Synthesis of Cu_2_O Octahedra and Their Morphology-Dependent Photocatalytic Properties. J. Phys. Chem. B.

[20] Prabhakaran G., Murugan R. (2012). Synthesis of Cu_2_O microcrystals with morphological evolution from octahedral to microrod through a simple surfactant-free chemical route. CrystEngComm.

[21] Periasamy A.P., Liu J., Lin H.-M., Chang H.-T. (2013). Synthesis of copper nanowire decorated reduced graphene oxide for electro-oxidation of methanol. J. Mater. Chem. A.

[22] Zhang W., Wen X., Yang S., Berta Y., Wang Z.L. (2003). Single-Crystalline Scroll-Type Nanotube Arrays of Copper Hydroxide Synthesized at Room Temperature. Adv. Mater..

[23] Kuo C.-H., Huang M.H. (2008). Fabrication of Truncated Rhombic Dodecahedral Cu_2_O Nanocages and Nanoframes by Particle Aggregation and Acidic Etching. J. Am. Chem. Soc..

[24] Wei S.Z., Kibsgaard J., Dickens C.F., Chorkendorff I.B., Nørskov J.K., Jaramillo T.F. (2017). Combining Theory and Experiment in Electrocatalysis: Insights into Materials Design. Science.

[25] Klinkova A., De Luna P., Dinh C.T., Voznyy O., Larin E.M., Kumacheva E., Sargent E.H. (2016). Rational Design of Efficient Palladium Catalysts for Electroreduction of Carbon Dioxide to Formate. ACS Catal..

[26] Hwee C., Ng B., Fan W.Y. (2006). Shape Evolution of Cu_2_O Nanostructures via Kinetic and Thermodynamic Controlled Growth. J. Phys. Chem. B.

[27] Basiratnia A., Rempel J., Li F., Pogodaev A., Zienchuk T.A., Klinkova A. (2019). Cu(II)-nanoparticle-derived structures under CO_2_ reduction conditions: A matter of shape. Phys. Chem. Chem. Phys..

[28] De Yoreo J.J., Gilbert P.U.P.A., Sommerdijk N.A.J.M., Penn R.L., Whitelam S., Joester D., Zhang H., Rimer J.D., Navrotsky A., Banfield J.F. (2015). Crystallization by Particle Attachment in Synthetic, Biogenic, and Geologic Environments. Science.

[29] Masoomi M.Y., Morsali A. (2013). Morphological Study and Potential Applications of Nano Matal-Organic Coordination Polymers. RSC Adv..

[30] Liu K., Shen Z.-R., Li Y., Han S.-D., Hu T.-L., Zhang D.-S., Bu X.-H., Ruan W.-J. (2014). Solvent Induced Rapid Modulation of Micro/Nano Structures of Metal Carboxylates Coordination Polymers: Mechanism and Morphology Dependent Magnetism. Sci. Rep..

[31] Duan H., Wang D., Li Y. (2015). Green chemistry for nanoparticle synthesis. Chem. Soc. Rev..

[32] Littrell D.M., Bowers D.H., Tatarchuk B.J. (1987). Hydrazine reduction of transition-metal oxides. J. Chem. Soc. Faraday Trans. 1.

[33] Djokić S.S. (1997). Electroless Deposition of Cobalt Using Hydrazine as a Reducing Agent. J. Electrochem. Soc..

[34] Eluri R., Paul B. (2012). Synthesis of nickel nanoparticles by hydrazine reduction: Mechanistic study and continuous flow synthesis. J. Nanopart. Res..

[35] LaGrow A.P., Sinatra L., Elshewy A., Huang K.W., Katsiev K., Kirmani A.R., Amassian A., Anjum D.H., Bakr O.M. (2014). Synthesis of Copper Hydroxide Branched Nanocages and Their Transformation to Copper Oxide. J. Phys. Chem. C.

[36] Cai R., Yang D., Peng S., Chen X., Huang Y., Liu Y., Hou W., Yang S., Liu Z., Tan W. (2015). Single Nanoparticle to 3D Supercage: Framing for an Artificial Enzyme System. J. Am. Chem. Soc..

[37] Heaton B.T., Jacob C., Page P. (1996). Transition metal complexes containing hydrazine and substituted hydrazines. Coord. Chem. Rev..

[38] Brown D.B., Donner J.A., Hall J.W., Wilson S.R., Wilson R.B., Hodgson D.J., Hatfield W.E. (1979). Interaction of hydrazine with copper(II) chloride in acidic solutions. Formation, spectral and magnetic properties, and structures of copper(II), copper(I), and mixed-valence species. Inorg. Chem..

[39] Nicholls D., Swlndells R. (1969). Hydrazine complexes of copper(I) chloride. J. Inorg. Nucl. Chem..

[40] Sui Y., Fu W., Yang H., Zeng Y., Zhang Y., Zhao Q., Li Y., Zhou X., Leng Y., Li M. (2010). Low Temperature Synthesis of Cu_2_O Crystals: Shape Evolution and Growth Mechanism. Cryst. Growth Des..

[41] Su D.S., Zhang B., Schlögl R. (2015). Electron Microscopy of Solid Catalysts—Transforming from a Challenge to a Toolbox. Chem. Rev..

[42] Nakamoto K. (2008). Infrared and Raman Spectra of Inorganic and Coordination Compounds: Part A: Theory and Applications in Inorganic Chemistry.

[43] Jirgensons B. (1952). Solubility and fractionation of polyvinylpyrrolidone. J. Polym. Sci..

