# Supplementary material for: Reductive and Coordinative Effects of Hydrazine in Structural Transformations of Copper Hydroxide Nanoparticles
Source: Nanomaterials (Basel). 2019 Oct 11;9(10):1445. doi: 10.3390/nano9101445 (PMC6835711; doi:10.3390/nano9101445)
Supplement: Supplementary file 1 [file nanomaterials-09-01445-s001.pdf]

nanomaterials

Supplementary Materials

Reductive and coordinative effects of hydrazine in structural transformations of copper hydroxide nanoparticles

Xenia Medvedeva [1], Aleksandra Vidyakina [1], Feng Li [1], Andrey Mereshchenko [1,2] and Anna Klinkova [1,*]

1 Department of Chemistry and Waterloo Institute for Nanotechnology, University of Waterloo, Waterloo, ON N2L 3G1, Canada; kseniia.azarova@uwaterloo.ca (X.M.); vidyakina.aleksandra@mail.ru (A.V.); feng.li@uwaterloo.ca (F.L.); a.mereshchenko@spbu.ru (A.M.)
2 Institute of Chemistry, Saint-Petersburg State University, 7/9 Universitetskaya emb., St. Petersburg 199034, Russia
* Correspondence: aklinkova@uwaterloo.ca

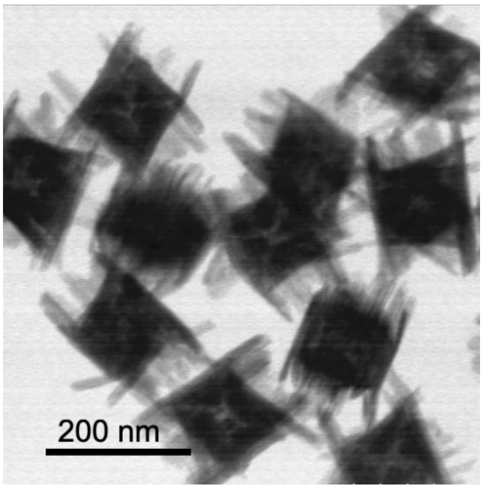

Figure S1. TEM image of as synthesized $Cu(OH)_2$ nanocages.

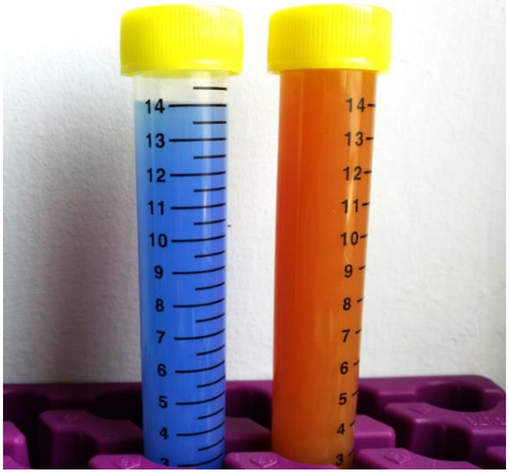

Figure S2. A photograph of $Cu(OH)_2$ nanocages colloid before (left) and after (right) introducing N_2H_4. Note that the color of hydrazine-treated solutions was the same at a stoichiometric N_2H_4 amount and excess N_2H_4 added to $Cu(OH)_2$ nanocages.

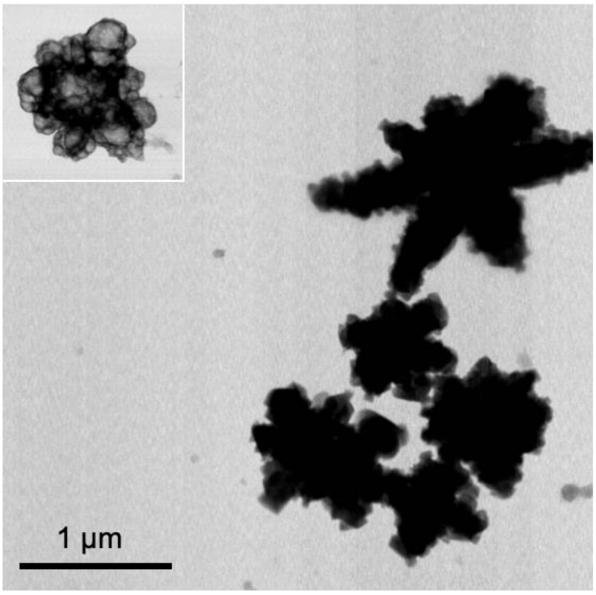

Figure S3. TEM image of Cu_2O-N_2H_4 obtained at $[N_2H_4]$:$[Cu(OH)_2]$ = 5 at 25kV accelerating voltage at 15,000× magnification. Inset shows the effect of electron beam exposure obtained at 25kV accelerating voltage and 70,000× magnification after 30 seconds exposure time (scale in the inset matches the figure).

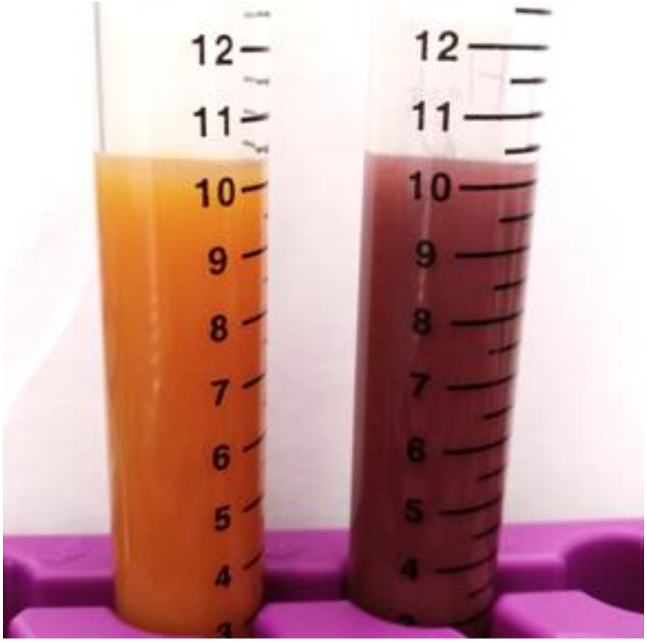

Figure S4. A photograph of Cu_2O-N_2H_4 colloid (obtained by treating $Cu(OH)_2$ nanocages in ethanol with hydrazine ($[N_2H_4]$:$[Cu(OH)_2]$ = 5) immediately after hydrazine addition (left) and after aging the mixture for 2 days (right).

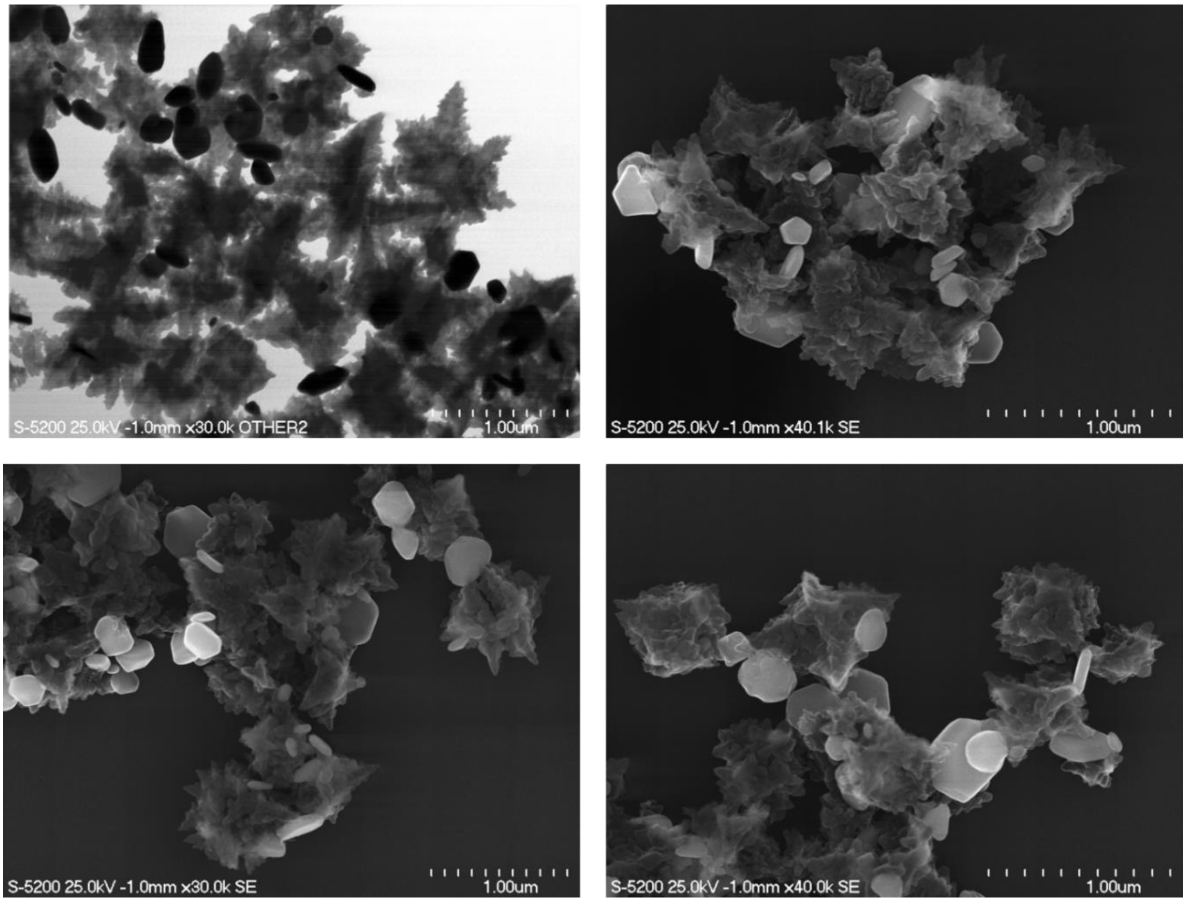

Figure S5. TEM (top left) and SEM (top right and bottom) images of Cu_2O-N_2H_4 colloid (obtained by treating $Cu(OH)_2$ nanocages in ethanol with hydrazine ($[N_2H_4]$:$[Cu(OH)_2]$ = 5) after its aging for 2 days.

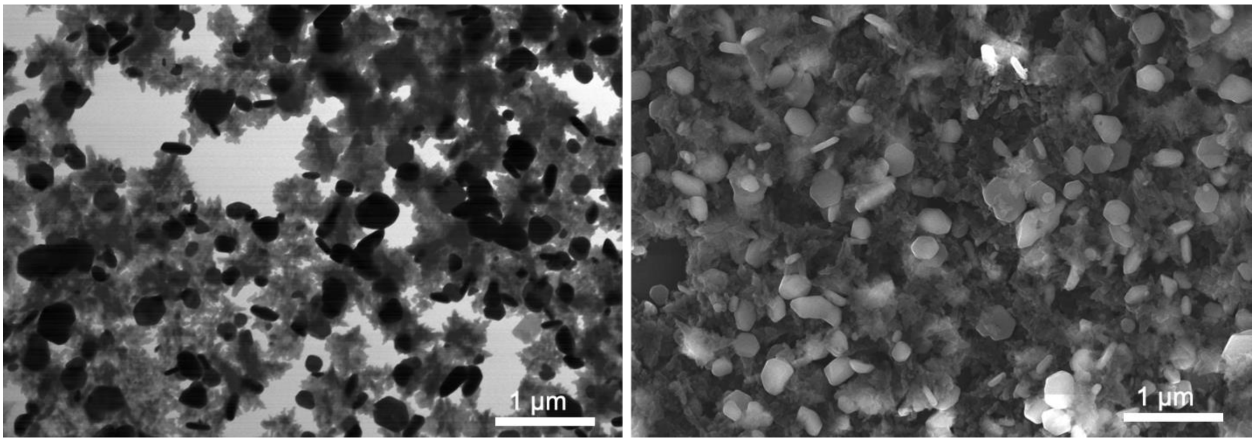

Figure S6. TEM (left) and SEM (right) images of Cu_2O-N_2H_4 colloid (obtained by treating $Cu(OH)_2$ nanocages in ethanol with hydrazine ($[N_2H_4]$:$[Cu(OH)_2]$ = 5) after its aging for 5 days.

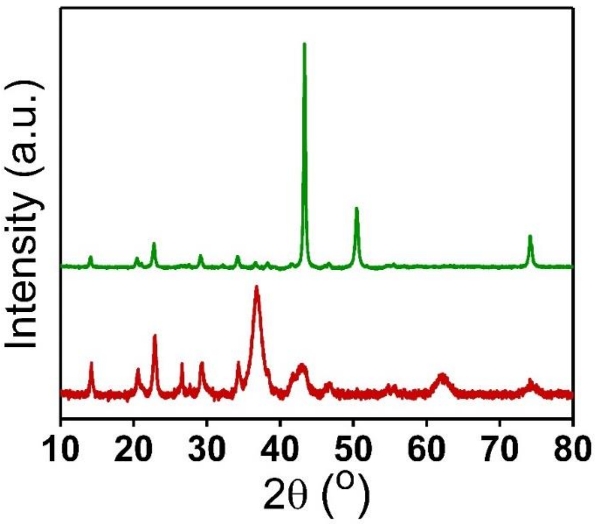

Figure S7. XRD patterns of Cu_2O-N_2H_4 colloid (obtained by treating $Cu(OH)_2$ nanocages in ethanol with hydrazine ($[N_2H_4]:[Cu(OH)_2] = 5$) within a day of hydrazine addition (red) and after aging the mixture for 5 days (green).

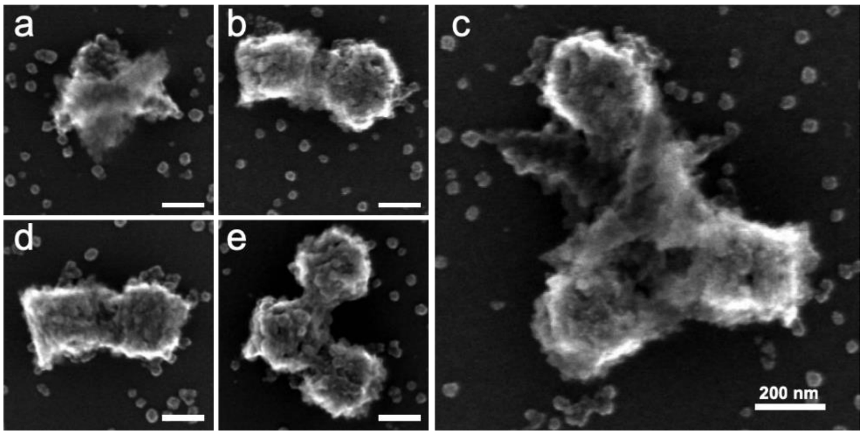

Figure S8. SEM images of the intermediate structures observed upon treatment of $Cu(OH)_2$ with 5-fold excess hydrazine compared to the stoichiometric ratio ($[N_2H_4]:[Cu(OH)_2] = 1.25$).

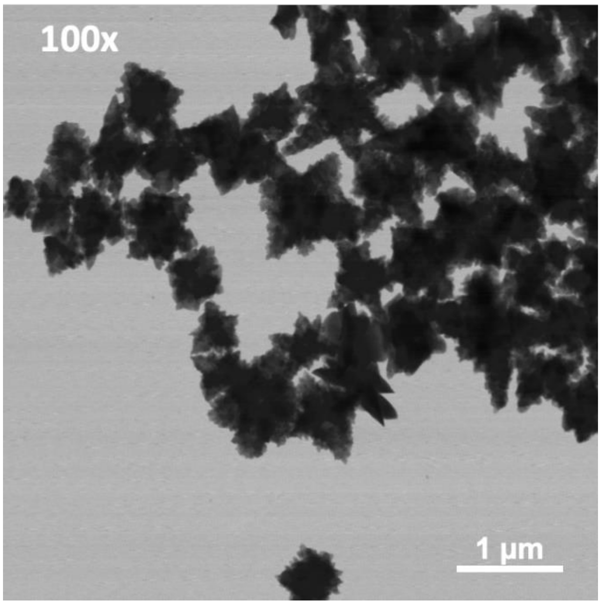

Figure S9. TEM image of the structures obtained from $Cu(OH)_2$ upon their reduction with 100-fold excess hydrazine compared to the stoichiometric ratio ($[N_2H_4]:[Cu(OH)_2]=25$).

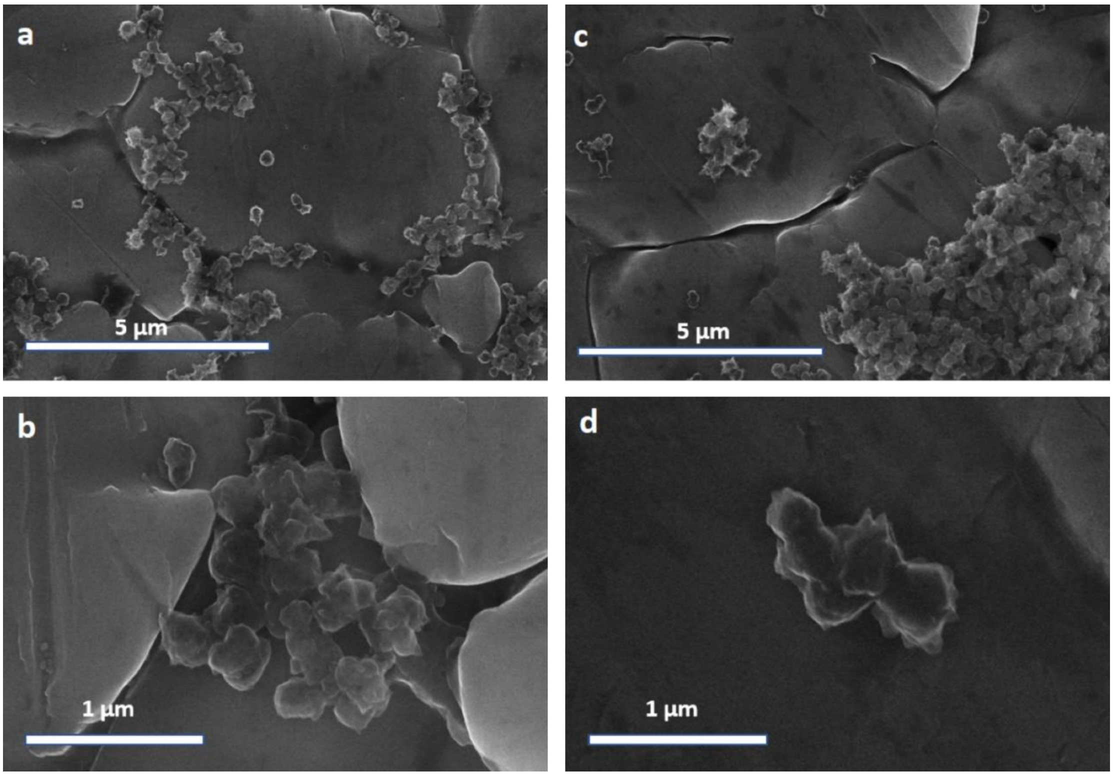

Figure S10. SEM images of the structures obtained by the reduction of $Cu(OH)_2$ nanocages with excess hydrazine ($[N_2H_4]:[Cu(OH)_2]=5$) in 1-propanol (**a**,**b**) and in 2-propanol (**c**,**d**).

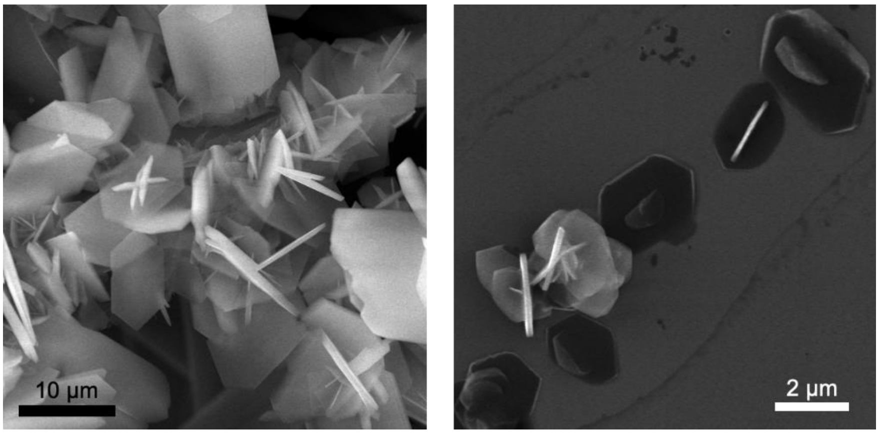

Figure S11. SEM images of the structures obtained by the reduction of $Cu(OH)_2$ nanocages with excess hydrazine ($[N_2H_4]:[Cu(OH)_2] = 5$) in water.

Composition calculations

All samples were obtained using ethanol as a solvent, and the powders for elemental analysis and XPS were prepared directly after the reactions. Specifically, the material was concentrated using centrifugation, the resulting precipitate was separated and dried in air for 1 h, followed by drying in a vacuum oven at 40 °C overnight.

Elemental analysis-based calculations were made assuming: (1) the samples contained only $Cu(OH)_2$, CuO/Cu_2O, PVP, EtOH and N_2H_4; (2) due to the same work-up procedure, all samples contained the same amount of PVP. The amount of PVP was calculated from the %N in the $Cu(OH)_2$ nanoframes containing no other sources of N. The fractions of N_2H_4 and EtOH were calculated from %N and %C, respectively, correcting for %PVP. The fraction of $Cu(OH)_2$ was calculated from the residual %H. The remainder was assumed to be CuO in the case of $Cu(OH)_2$ sample and Cu_2O in the reduced samples.

Table S1. Elemental analysis data: weight percentages (ω%) of N, C, and H in $Cu(OH)_2$ nanocages and structures obtained by their reduction with a stoichiometric amount and the excess of hydrazine ($[N_2H_4]:[Cu(OH)_2]$ 0, 0.25, and 5, respectively).

SAMPLE	$[N_2H_4]:[Cu(OH)]_2$	ω%N	ω%C	ω%H
$Cu(OH)_2$	0	0,38	5,54	2,37
“Cu_2O“	0.25	0,59	5,34	1,35
“Cu_2O-N_2H_4“	5	1,63	4,20	0,98

Table S2. Elemental analysis data converted to atomic percentages (a%) of N, C, and H, and calculated molar fractions (in%) of the components in $Cu(OH)_2$ nanocages and structures obtained by their reduction with a stoichiometric amount and the excess of hydrazine ($[N_2H_4]:[Cu(OH)_2]$ 0, 0.25, and 5, respectively).

SAMPLE	$[N_2H_4]:[Cu(OH)]_2$	a%N	a%C	a%H	%PVP	%EtOH	%N_2H_4	%$Cu(OH)_2$	%CuO	%Cu_2O
$Cu(OH)_2$	0	0,48	8.21	42.18	2.3	12.9	0	54.2	30.6	-
“Cu_2O“	0.25	1.06	11.14	33.83	3.2	16.8	0.9	14.2	-	64.9
“Cu_2O-N_2H_4“	5	3.36	10.1	28.31	3.4	11.7	5.6	0.01	-	79.3

Based on these molar fractions, up to 7% of Cu can be coordinated by –NH_2 of N_2H_4, and up to 10% of Cu can be coordinated by –OH of EtOH, in the sample obtained by the reduction of $Cu(OH)_2$ nanocages with 20-fold access hydrazine (i.e., $[N_2H_4]$:$[Cu(OH)_2]$-5).

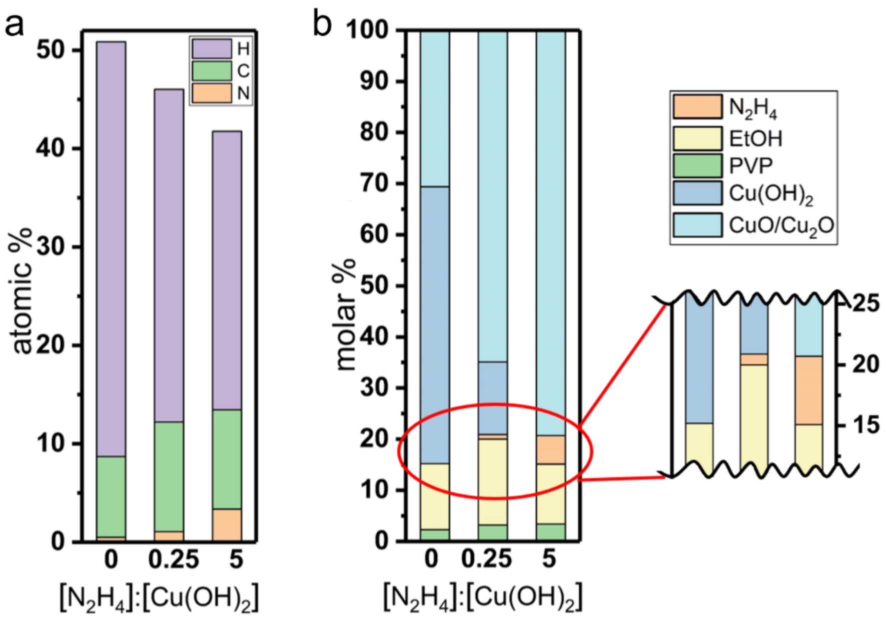

Figure S12. The composition of $Cu(OH)_2$ nanocages and the structures obtained upon their reduction with a stoichiometric amount and 20-fold excess of hydrazine ($[N_2H_4]$:$[Cu(OH)_2]$ 0, 0.25, and 5, respectively). (a) Atomic fractions of H, C, and N from CHN derived from the elemental analysis. (b) Molar fractions of the molecular components calculated from the elemental analysis assuming (1) the samples contained only $Cu(OH)_2$, CuO/Cu_2O, PVP, EtOH and N_2H_4, and (2) all three studied samples had the same amount of PVP (i.e., no loss if PVP upon hydrazine reduction).

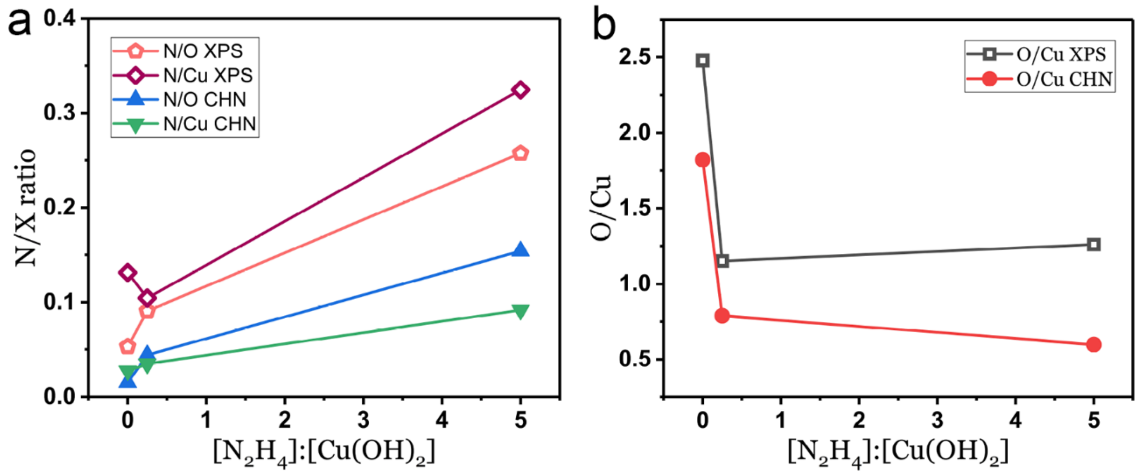

Figure S13. Comparison of the atomic ratios (N/O, N/Cu and O/Cu), calculated from the XPS and CHN analyses' data for the initial $Cu(OH)_2$ nanocages and structures obtained by the reduction thereof with stoichiometric and excessive amounts of hydrazine ($[N_2H_4]$:$[Cu(OH)_2]$ 0.25 and 5, respectively).

Table S3. Comparison of atomic percentages (a%) of Cu, O, and N, obtained from XPS analysis and calculated from the CHN data for $Cu(OH)_2$ nanocages and the structures obtained upon their reduction with a stoichiometric amount and 20-fold excess of hydrazine ($[N_2H_4]:[Cu(OH)_2]$ 0, 0.25, and 5, respectively).

**$[N_2H_4]:[Cu(OH)_2]$**	**XPS**			**CHN**		
	Cu 2p, a%	O 1s, a%	N 1s, a%	Cu, a%	O, a%	N, a%
**0**	27.71	68.65	3.64	35.1	63.93	0.97
**0.25**	44.34	51.03	4.63	54.86	43.23	1.91
**5**	38.69	48.76	12.55	59.24	35.32	5.44

Based on the CHN atomic fractions, no greater than 9% of Cu can be coordinated by $-NH_2$ of N_2H_4 in the sample obtained by the reduction of $Cu(OH)_2$ nanocages with 20-fold access hydrazine (i.e., $[N_2H_4]:[Cu(OH)_2] = 5$), even if no PVP was present in the sample (i.e., assuming that all detected N was associated with N_2H_4).

A higher [Cu]:[N] ratio in XPS analysis, 32, compared to 9 in CHN (in the samples obtained at $[N_2H_4]:[Cu(OH)_2] = 5$), supports that PVP is still present at the surface of the particles.
